# Polymorphism and Structural Variety in Sn(II) Carboxylate Coordination Polymers Revealed from Structure Solution of Microcrystals

**DOI:** 10.1002/smtd.202301703

**Published:** 2024-03-10

**Authors:** Avneet K. Ramana, Jeremiah P. Tidey, Geraldo M. de Lima, Richard I. Walton

**Affiliations:** ^1^ Department of Chemistry University of Warwick Coventry CV4 7AL UK; ^2^ Department of Physics University of Warwick Coventry CV4 7AL UK; ^3^ Departamento de Química Universidade Federal de Minas Gerais Avenida Antônio Carlos 6627 Belo Horizonte MG CEP 31270‐901 Brazil

**Keywords:** crystal structure, electron diffraction, MOFs, synchrotron

## Abstract

The crystal structures of four coordination polymers constructed from Sn(II) and polydentate carboxylate ligands are reported. All are prepared under hydrothermal conditions in KOH or LiOH solutions (either water or methanol–water) at 130−180 °C and crystallize as small crystals, microns or less in size. Single‐crystal structure solution and refinement are performed using synchrotron X‐ray diffraction for two materials and using 3D electron diffraction (3DED) for the others. Sn_2_(1,3,5‐BTC)(OH), where 1,3,5‐BTC is benzene‐1,3,5‐tricarboxylate, is a new polymorph of this composition and has a three‐dimensionally connected structure with potential for porosity. Sn(H‐1,3,5‐BTC) retains a partially protonated ligand and has a 1D chain structure bound by hydrogen bonding via ─COOH groups. Sn(H‐1,2,4‐BTC) contains an isomeric ligand, benzene‐1,2,4‐tricarboxylate, and contains inorganic chains in a layered structure held by hydrogen bonding. Sn_2_(DOBDC), where DOBDC is 2,5‐dioxido‐benzene‐1,4‐dicarboxylate, is a new polymorph for this composition and has a three‐dimensionally connected structure where both carboxylate and oxido groups bind to the tin centers to create a dense network with dimers of tin. In all materials, the Sn centers are found in highly asymmetric coordination, as expected for Sn(II). For all materials phase purity of the bulk is confirmed using powder X‐ray diffraction, thermogravimetric analysis, and infrared spectroscopy.

## Graphical abstract

A set of coordination polymers constructed from Sn(II) and polycarboxylate ligands crystallize under hydrothermal conditions as microcrystalline powders. Structure solution using either synchrotron X‐ray diffraction or 3D‐electron diffraction reveals complex structural chemistry and polymorphism in this family of materials, in part due to the asymmetric coordination preference of the 5s^2^ main group cation.

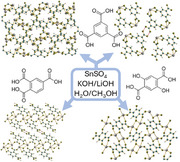

## 1 Introduction

Coordination polymers and metal‐organic frameworks of tin have been less studied than those of metals from other parts of the Periodic Table, such as the transition metals and the rare earths for which extensive families of materials have been synthesized, structurally characterized, and properties are beginning to be exploited.^[^ ^1^, ^2^, ^3^ ^]^ The use of tin as a metal center for the formation of coordination polymers is appealing since the metal can exist in two oxidation states, +2 and +4, each of which has useful characteristics.^[^ ^4^ ^]^ The chemistry of Sn(IV) gives Lewis acidic properties, which can be exploited in porous materials such as zeolites to give heterogeneous solid‐acid catalysts for important organic transformations.^[^ ^5^ ^]^ Sn(II) is expected to show highly unsymmetrical coordination environment due to the presence of the sterochemically active 5s^2^ pair of electrons and so would be anticipated to yield unusual and unique framework topologies. Usually Sn(II) is easily oxidized to Sn(IV), and so the higher oxidation state is found in many tin materials,^[^ ^6^ ^]^ but in coordination polymers constructed from carboxylates we have previously found water‐stable Sn(II) materials, prepared under hydrothermal conditions.^[^ ^7^ ^]^ Water‐stable coordination polymers and metal‐organic frameworks are highly desirable for materials that can be used in the presence of moisture, important for many practical applications, but many coordination polymers are unstable with respect to hydrolysis.^[^ ^8^, ^9^, ^10^ ^]^ For the Sn(II) materials the use of polydentate, anionic carboxylate linkers with aromatic cores is likely to contribute to their water stability, since strong metal–ligand bonds are present, with a rigid organic ligand that is hydrophobic. The water stability of a Sn(II)‐BTEC (BTEC = benzene‐1,2,4,5‐tetracarboxylate) coordination polymer was exploited for selective sensing of chromate(VI) in aqueous media.^[^ ^11^ ^]^ Other examples of Sn(II) carboxylates have been investigated for lithium storage in batteries by Liu et al., who used the materials Sn_2_(DOBDC) (DOBDC = 2,5‐dioxido‐benzene‐1,4‐dicarboxylate) and Sn_2_(DOBPDC) (DOBPDC = 4,4′‐dioxidobiphenyl‐3,3′‐dicarboxylate) as hosts for lithium storage via the reversible formation of coordination bonds.^[^ ^12^ ^]^ This work was recently extended to further examples, where the ligand aromaticity was found to play an important role in charge transfer during lithium uptake,^[^ ^13^, ^14^, ^15^ ^]^ and by studying structures constructed from isomers of ligands, kinetics of lithium transport could be optimized.^[^ ^16^ ^]^ For the material Sn[(PDC)(H_2_O)] (PDC = pyridine‐2,6‐dicarboxylate) it was shown to dehydrate to an anhydrous form Sn[(PDC)], with noncentrosymmetric crystal structure maintained and both materials having measurable second harmonic generation properties.^[^ ^17^ ^]^

Here we have examined the crystallization of Sn(II) carboxylates systematically, exploring synthesis conditions and investigating the use of new ligands, leading to new polymorphs and new materials. As with many coordination polymers and metal‐organic frameworks, the materials do not typically form as large enough crystals for conventional laboratory X‐ray diffraction, and we have used synchrotron radiation and electron diffraction to resolve crystal structures from specimens only microns in size to allow accurate description of their structures.

## 2 Results and Discussion

The synthesis of four materials was achieved under hydrothermal conditions in KOH solution with SnSO_4_ as tin precursor and using the carboxylic acids benzene‐1,3,5‐tricarboxylic acid (H_3_‐1,3,5‐BTC), benzene‐1,2,4‐tricarboxylic acid (H_3_‐1,2,4‐BTC) and 2,5‐dihydroxy‐benzene‐1,4‐dicarboxylic acid (H_4_‐DOBDC). The full details are presented below in the Experimental Section. The first linker has previously been used in earlier work for forming Sn(II) coordination polymers,^[^ ^7^, ^14^ ^]^ but we explored solvent and reaction time to establish the phase selectivity of synthesis conditions. The second ligand to our knowledge has not yet been explored for Sn(II) coordination polymers. The last ligand had been used for construction of a Li‐storage host but prepared using different reagents than we have used, so we examined the choice of precursors in synthesis, and in doing so isolated a new polymorph. **Table** **1** contains crystallographic data for the 4 materials characterized using single‐crystal diffraction methods. Images of the crystals studied by 3DED are provided in Figures S1 and S2 (Supporting Information).

**Table 1:** Crystallographic data for the four materials studied.

	Sn_2_(1,3,5‐BTC)(OH)	Sn(H‐1,3,5‐BTC)	Sn(H‐1,2,4‐BTC)	Sn_2_(DOBDC)
Empirical formula	C_9_H_4_O_7_Sn_2_	C_9_H_4_O_6_Sn	C_9_H_4_O_6_Sn	C_4_HO_3_Sn
Crystal system	Orthorhombic	Triclinic	Monoclinic	Monoclinic
Space group	*Pna*2_1_	*P* $\bar{1}$	*C*2/*c*	*P*2_1_/*c*
*a* [Å]	10.7763(5)	4.5534(3)	13.90(9)	4.98(7)
*b* [Å]	12.4014(6)	9.8583(7)	4.87(10)	11.05(5)
*c* [Å]	7.9574(3)	10.3440(8)	25.65(19)	7.92(10)
*α* [°]	90	72.621(7)	90	90
*β* [°]	90	84.440(6)	97.33(16)	104.6(6)
*γ* [°]	90	79.459(6)	90	90
*V* [Å^3^]	1063.44(8)	435.21(6)	1722(39)	422(8)
*Z*	4	2	8	2
Data collection	Synchrotron X‐ray	Synchrotron X‐ray	3DED	3DED
Wavelength [Å]	0.68890	0.68890	0.02510	0.02510
*T* [K]	100	100	150	150
Number of datasets merged	1	1	3	4
Dimensions of crystal(s) [µm^3^]	50×50×50	50×20×20	0.5×0.2×0.1 0.4×0.3×0.2 1.0×0.8×0.2	0.8×0.3×0.2 0.8×0.5×0.4 0.8×0.7×0.3 1.0×0.5×0.4
*µ* [mm^−1^]	4.372	2.728	n/a	n/a
Index ranges	−17≤*h*≤17 −19≤*k*≤20 −12≤*l*≤12	−7≤*h*≤7 −15≤*k*≤15 −16≤*l*≤15	−6≤*h*≤6 −13≤*k*≤13 −9≤*l*≤9	−17≤*h*≤17 −6≤*k*≤6 −32≤*l*≤32
Reflections collected	30679	10179	9840	6383
Independent reflections	4451	3368	1760	683
*Completeness to 0.80 Å*	100	99.0	99.5%	79.4%
Data/restraints/parameters	4327/2/167	2949/0/145	1498/244/153	655/54/74
Goodness‐of‐fit on *F* ^2^	1.078	1.072	1.6946	1.3145
Final *R1* [*Fo* > 4*σ*(*Fo*)]	0.0236	0.0464	0.1608	0.1410
*Final R1/wR2* (all data)	0.0248/0.0525	0.0529/0.1180	0.1782/0.4287	0.1456/0.3644
Δ*ρ*_max_ and Δ*ρ*_min_ [e Å^−3^]	0.999/−0.654	2.662/−3.443	1.6089/−0.9254	0.7081/−0.7658
CCDC reference	2309733	2309734	2309735	2309736

### 2.1 Sn_2_(1,3,5‐BTC)(OH)

**Figure 1:**
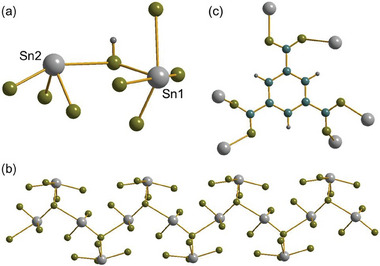
Structural units in Sn_2_(1,3,5‐BTC)(OH). a) The local oxygen environment of the two unique tin centers, linked by the oxygen of a bridging hydroxide, b) the infinite inorganic chains running parallel to *c* (protons not shown), and c) the bonding of the BTC ligand to tin centers. Sn is gray, carbon blue, hydrogen dark gray, and oxygen olive.

The structure of this material was solved and refined from synchrotron X‐ray data, and is a new polymorph of a composition previously reported by our group.^[^ ^7^ ^]^ The previous material was prepared in aqueous KOH solution but, here, LiOH in a water–methanol mixture led to the formation of the second polymorph. The material crystallizes in the orthorhombic space group, *Pna*2_1_, and has a three‐dimensionally connected structure with potential void space, hence, can be classed as a metal‐organic framework. The unit cell contains two unique Sn(II) cations and a benzene‐1,3,5‐tricarboxylate ligand. The Sn─O bond distances range from 2.140(2) to 2.386(3) Å, with a second set of more distant contacts in the range 2.83 to 3.13 Å (see **Figure** **1**a,b), being comparable to those previously reported for Sn(II) carboxylates.^[^ ^7^ ^]^ Sn1 has a disphenoidal geometry with four coordinated oxygens. The smallest bond angle is O10─Sn1─O11 is 76.38(9) ° and the largest bond angle is O52─Sn1─O32 is 157.8(1) °, while Sn2 has a trigonal pyramidal structure and is bonded to three oxygens. The tin centers are linked together by a hydroxide (for which the proton is identified from the difference map) that triply bridges two Sn2 and one Sn1, Figure 1c. The assignment of the bridging oxygen as a hydroxide anion was also confirmed using the bond valence sum method.^[^ ^18^ ^]^ The bond angle of Sn1─O10A─Sn2 is 117.79 °, O52─Sn1─O10A is 79.40 °, and O51─Sn2─O10A is 84.67 °. The bond angles of O─Sn─O range from 76.36° to 157.79°. The tin centers are connected to give infinite inorganic chains [─Sn2─(OH)─Sn2─] with pendant Sn1 centers, Figure 1b, that run parallel to *c*. The remaining oxygens that complete the coordination of tin come from the BTC ligand, and each of the carboxylate groups binds in a *Z*,*Z*‐µ_2_‐η^1^:η^1^ manner, such that each oxygen connects to a different Sn center (Figure 1c).

**Figure 2:**
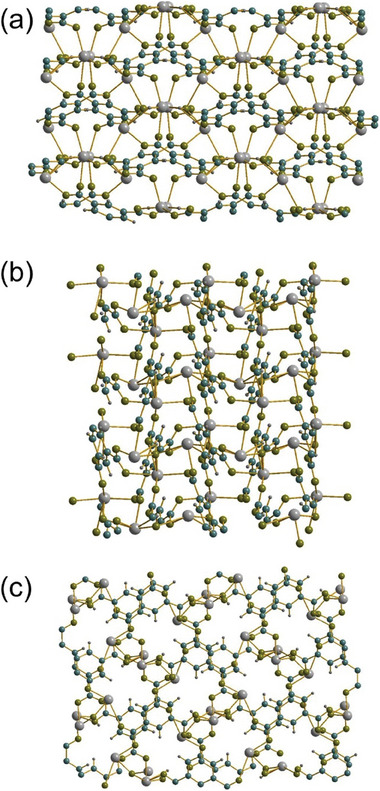
The structure of Sn_2_(1,3,5‐BTC)(OH) viewed along a) *a*, b) *b*, and c) *c*. Sn is gray, carbon blue, hydrogen dark gray, and oxygen olive.

The 1,3,5‐BTC ligands cross‐link the chain motifs to yield a three dimensionally connected structure. Using the notation of Cheetham et al.,^[^ ^19^ ^]^ the network has connectivity I^1^O^2^. **Figure** **2** shows projections of the structure in different directions. While the structure is densely packed in the *ac* and *bc* planes, potential porosity can be seen when viewing the *ab* plane, Figure 2c, with channels running parallel to *c* having a cross‐sectional diameter defined by atomic distances in excess of 4 Å (Sn─O = 4.062(3) Å and Sn─Sn = 4.9923(4) Å). However, these do not constitute truly accessible voids: a probe size of <0.6 Å required to obtain continuous channels and using >0.93 Å  presents no void space, using the hard‐sphere probe approach employed using Mercury (version, 2023.3.0, Build 364735).^[^ ^20^ ^]^ Similarly, only 5.27 Å^3^ void per unit cell, distributed across four distinct pockets, is found using an isovalue as high as 0.001 e Å^−3^ with the Independent Atoms in Molecules (IAM) type electron density calculation employed by CrystalExplorer.^[^ ^21^ ^]^

**Figure 3:**
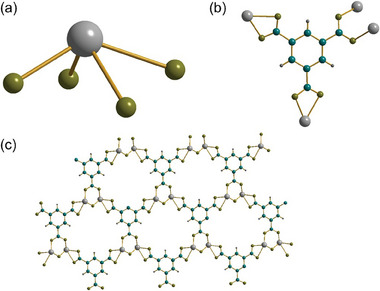
The structure of the previously reported polymorph of Sn_2_(1,3,5‐BTC)(OH).^[^ ^7^ ^]^ a) The local environment of the single, b) the binding mode of the BTC ligand and c) view of part of the infinite layers. Sn is grey, carbon blue, hydrogen dark gray, and oxygen olive.

The new structure of Sn_2_(1,3,5‐BTC)(OH) reported here is strikingly different from the previously reported polymorph, for which tin is only found in a single, rather more distorted four‐coordinate disphenoidal site, **Figure** **3**a, which approaches a square‐based pyramidal geometry. These are held in dimers that are bridged by the hydroxide ion, and the 1,3,5‐BTC ligands has carboxylates that bind in two different modes: one bridging pairs of tins (*Z*,*Z*‐µ_2_‐η^1^:η^1^) and two each chelating a single tin (η^2^), Figure 3b.^[^ ^7^ ^]^ The overall structure extends in two dimensions, bound only by bridging ligands and no infinite inorganic connectivity so is classed as an I^0^O^2^ structure, Figure 3c.

**Figure 4:**
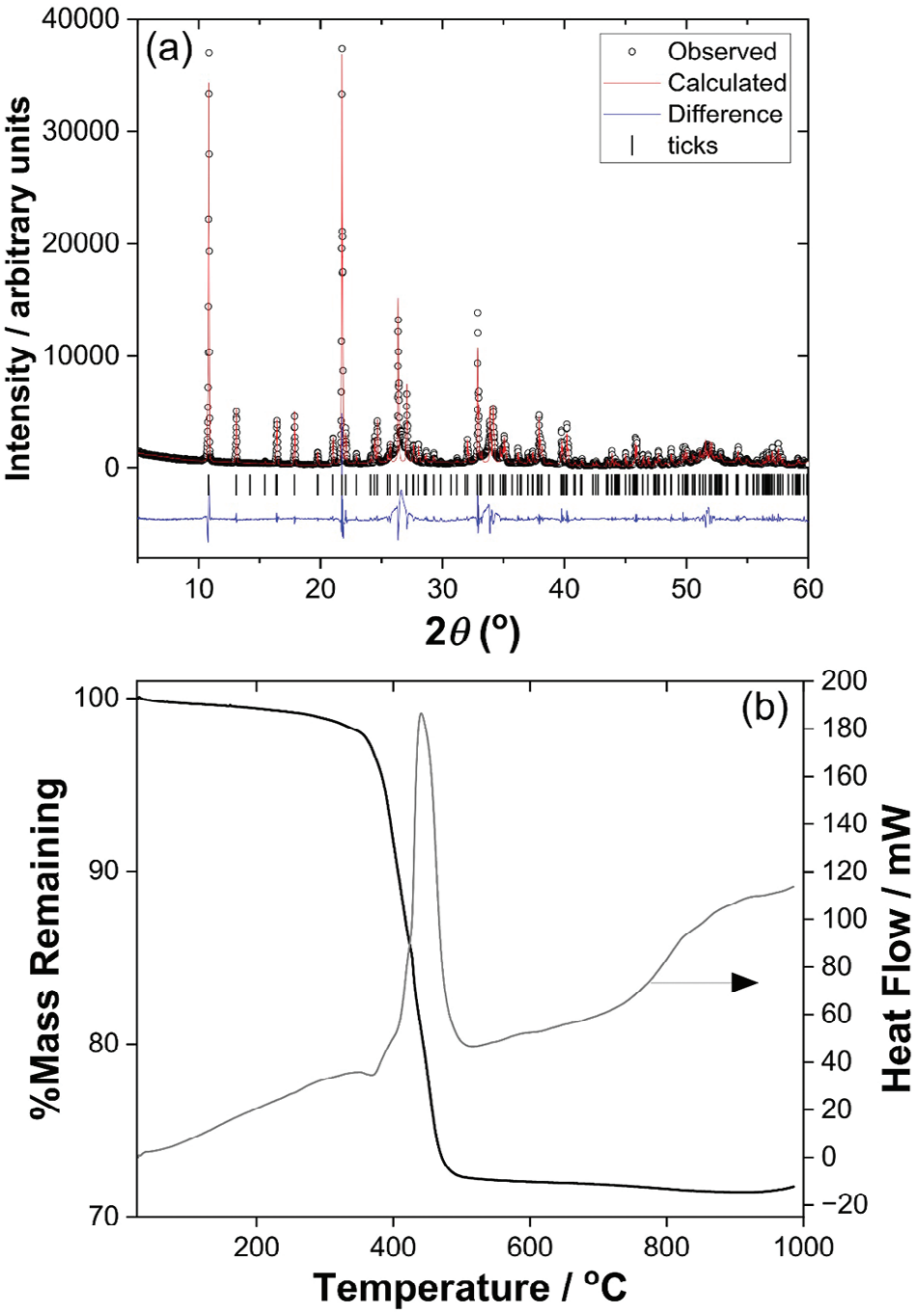
Characterization of the bulk sample of Sn_2_(1,3,5‐BTC)(OH). a) Pawley fitted powder XRD (*λ* = 1.5418 Å, see Supporting Information for fitted lattice parameters) and b) TGA‐DSC.

**Figure** **4** shows powder XRD and TGA from the bulk sample of Sn_2_(1,3,5‐BTC)(OH) to test the phase purity of the material. The powder XRD pattern, Figure 4a, shows clear evidence of preferred orientation in the sample, and the broad background features at ≈27 and 34°2*θ* correspond to the two strongest Bragg peaks of rutile‐type SnO_2_.^[^ ^22^ ^]^ The TGA, Figure 4b, shows the material to have reasonable thermal stability, with the most significant decomposition not occurring until above 400 °C, following some loss of surface solvent at lower temperatures. The theoretical mass loss was calculated to be 34.68%, while the experimental result showed the mass loss to be lower at 28.23%, which is likely due to the presence of the small amount of SnO_2_ in the sample. The infrared spectrum of Sn_2_(1,3,5‐BTC)(OH) shows a sharp O─H stretching band at ≈3490 cm^−1^ (Figure S3, Supporting Information), while in the carboxylate region the asymmetric and symmetric vibrations of coordinated carboxylates are observed at ≈1610 and ≈1420 cm^−1^, respectively, and there is no evidence of free carboxylic acid groups (Figure S4, Supporting Information), all of which are entirely consistent with the formulation of the material deduced from crystallography.

### 2.2 Sn(H‐1,3,5‐BTC)

Sn(H‐1,3,5‐BTC) crystallizes in the triclinic *P* $\bar{1}$ space group and the unit cell contains a unique Sn(II) cation, and a benzene‐1,3,5‐tricarboxylate ligand that is monoprotonated, with the proton clearly located in the Fourier difference map. Despite the fact that the synthesis of this material uses a higher hydroxide concentration compared to use for Sn_2_(1,3,5‐BTC)(OH), the acid precursor of the ligand retains one of its protons. During the course of our work, Jiang et al. recently reported the same material prepared using a different synthesis method (80 °C for 48 h in water alone).^[^ ^14^ ^]^

**Figure 5:**
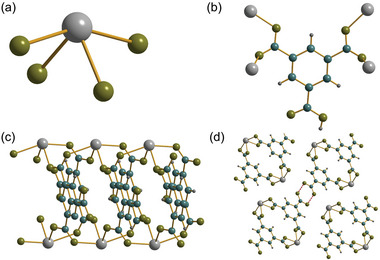
Views of the of structure of Sn(H‐1,3,5‐BTC). a) The local coordination environment of Sn, b) the binding mode of the HBTC ligand, c) connectivity of the structure showing one of the tubular chains running parallel to *a*, and d) projection in the *bc* plane showing the stacking of the chains with the interchain hydrogen bonding interaction shown as the red dotted line. Sn is gray, carbon blue, hydrogen dark gray, and oxygen olive.

In Sn(H‐1,3,5‐BTC) Sn(II) is bonded to four different oxygen atoms and has a disphenoidal geometry similar to that of Sn1 for Sn_2_(1,3,5‐BTC)(OH), **Figure** **5**a. The Sn‐O distances range from 2.136(3) Å to 2.506(3) Å, as expected by comparison with other Sn(II) coordination polymers. The smallest bond angle is O1─Sn─O3 is 79.52(11) ° and the largest bond angle is O2─Sn─O4 is 155.89(10)°. The 1,3,5‐BTC ligand bonds through two of the carboxylate groups, to two pairs of tins, each in a *Z*,*Z*‐µ_2_‐η^1^:η^1^ binding mode, Figure 5b, while the third carboxylate group is not coordinated to any tin center. Instead, this is engaged in mutual hydrogen bonding with its inversion equivalent and the proton is clearly located in the difference map. The structure is connected to give tubular chains running along a, Figure 5c, which are held together by the hydrogen bonds in the *bc* plane between the ‐COOH groups, Figure 5d. The material is therefore classed as a 1D coordination polymer with overall connectivity I^0^O^1^.

**Figure 6:**
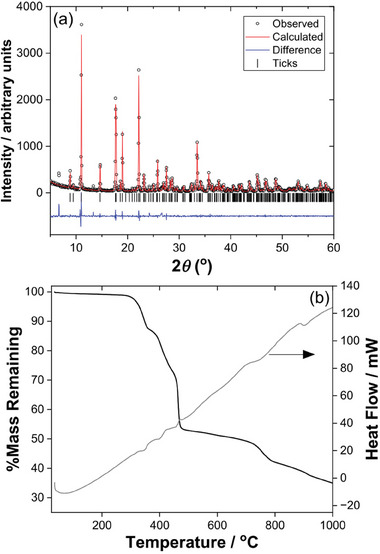
Characterization of the bulk sample of Sn(H‐1,3,5‐BTC). a) Pawley fitted powder XRD (*λ* = 1.5418 Å, see Supporting Information for fitted lattice parameters) and b) TGA‐DSC.

Powder XRD of Sn(H‐1,3,5‐BTC), **Figure** **6**a, shows good agreement in terms of peak positions with no evident impurity peaks present, although the relative peak intensities are evidently severely affected by preferred orientation, which is consistent with the needle‐like morphology of the crystallites. The TGA, Figure 6b, shows a complex set of mass loss events. The total theoretical mass loss is calculated to be 53.89%, assuming combustion to yield SnO_2_, while the experimental result shows the mass loss to be higher at 59.15%. This can be attributed small traces of linker still present in the powder or additional solvent due to incomplete drying. The infrared spectrum of Sn(H‐1,3,5‐BTC) shows the presence of broad O─H stretching bands in the region 2500–3300 cm^−1^, assigned as due to the carboxylic group (Figure S3, Supporting Information). In the carboxylate region the asymmetric and symmetric vibrations of coordinated carboxylates are observed at ≈1610 and ≈1420 cm^−1^, respectively, but also a distinct C═O stretch due to the carboxylic group at ≈1690 cm^−1^ (Figure S4, Supporting Information), which is notably absent in the IR spectrum of Sn_2_(1,3,5‐BTC)(OH) discussed above.

### 2.3 Sn(H‐1,2,4‐BTC)

**Figure 7:**
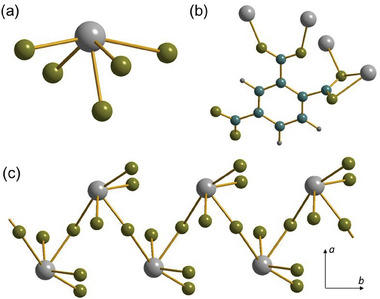
Views of the of structure of Sn(H‐1,2,4‐BTC). a) the local coordination environment of Sn, b) the binding mode of the 1,2,4‐BTC ligand, c) the infinite inorganic chain, with carbons and hydrogens removed for clarity. Sn is gray, carbon blue, hydrogen dark gray, and oxygen olive.

Sn(H‐1,2,4 BTC) contains a ligand not previously used to prepare Sn(II) coordination polymers and using a synthesis method similar to our earlier work for this type of material, we found it crystallizes in the monoclinic *C*2/*c* space group. In the absence of any single crystals suitable for X‐ray characterization, the structure was solved using continuous rotation 3DED, after grinding the sample and dispersing as a solid onto a standard graphene oxide TEM grid. Data treatment is described in the experimental section and follows current standard kinematical procedures currently available in the software CrysAlisPro (Oxford Diffraction /Agilent Technologies UK Ltd, Yarnton, England) and Olex2.^[^ ^23^ ^]^ The unit cell contains a unique Sn(II) cation which is bonded to five oxygen atoms with a distorted pyramidal coordination geometry, **Figure** **7**a. The bond angle of O1─Sn─O2 is 55.60 °. Four Sn─O distances range from 2.202(18) to 2.435(16) Å with one more distant interaction at 2.725(16) Å. The ligand contains one carboxylate group that remains protonated and one that is bonded to a pair of tin centers in a *Z*,*Z*‐µ_2_‐η^1^:η^1^ fashion, while the third is bonded to a pair of tin centers in a µ_2_‐η^2^:η^1^ manner (Figure 7b). The tin centers are linked by via the long Sn─O bonds to give a zig‐zag inorganic chain that runs parallel to *b*. (Figure 7c). The chains are cross‐linked by bridging carboxylates in the *a* direction to yield the 3D structure: this gives dimers of Sn centers bridged by Sn─O─C─O─Sn connections (*Z*,*Z*‐µ_2_‐η^1^:η^1^).

**Figure 8:**
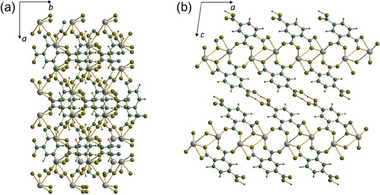
Views of the of structure of Sn(H‐1,2,4‐BTC). a) The infinite layers in the *ab* plane, b) stacking of the layers with interlayer hydrogen bonding indicated by dotted lines. Sn is gray, carbon blue, hydrogen dark gray, and oxygen olive.

The ligand connects the inorganic chains to give a layer in the *ab* plane of the crystal structure, **Figure** **8**a. The layers are held together by hydrogen bonds between the neighboring ─COOH groups of the 1,2,4‐BTC ligands, Figure 8b. The closest O‐O distance between the neighboring carboxylate groups is 2.630(16) Å, which confirms the hydrogen bonding interaction. The structure can be classed as a 2D coordination polymer, with connectivity I^1^O^1^

**Figure 9:**
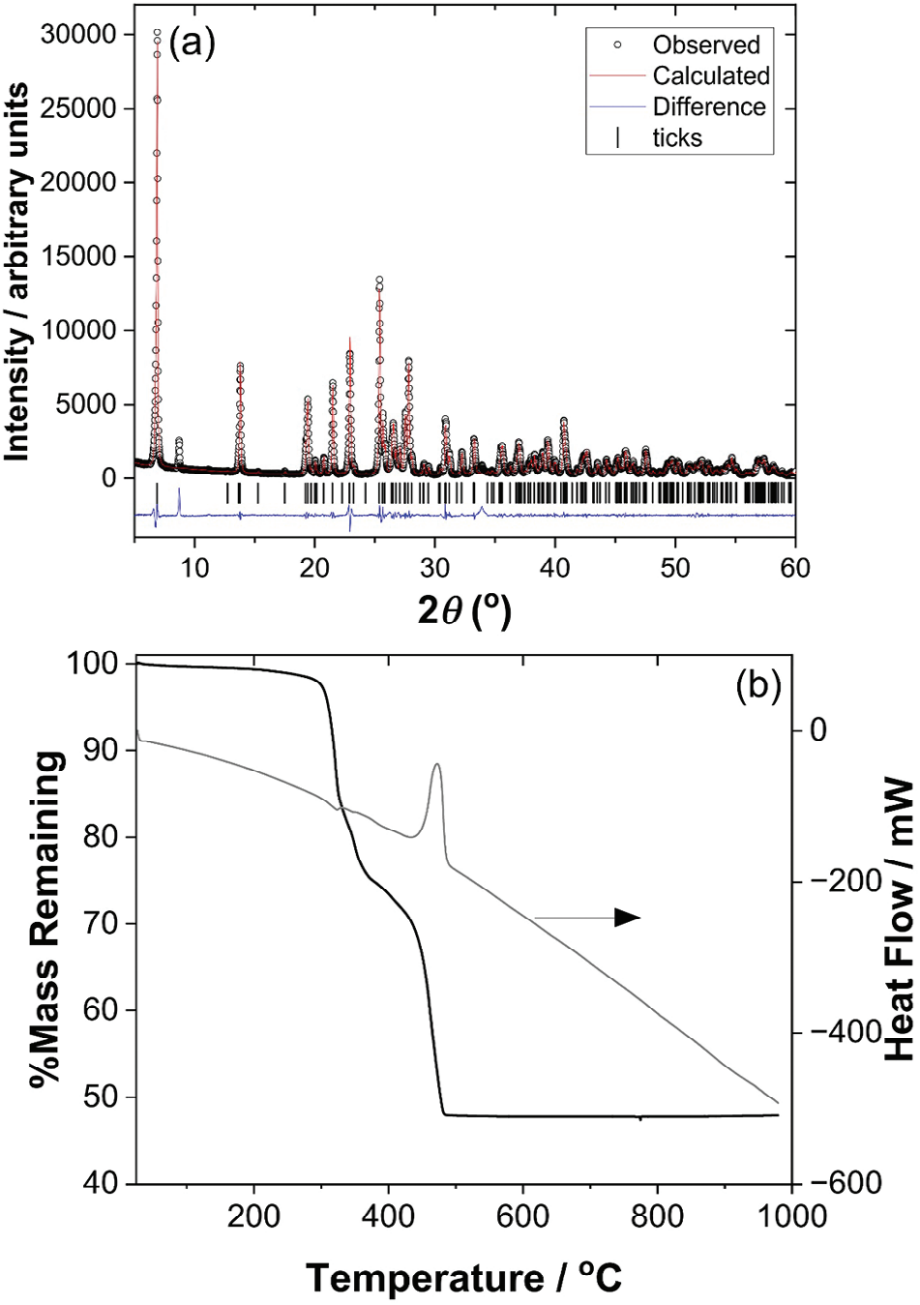
Characterization of the bulk sample of Sn(H‐1,3,5‐BTC). a) Pawley fitted powder XRD (*λ* = 1.5418 Å, see Supporting Information for fitted lattice parameters) and b) TGA.

**Figure** **9**a shows powder XRD, which shows good agreement between the measured and simulated patterns, with evidence of a minor impurity with strongest reflection observed at ≈9 °2*θ*. TGA, Figure 9b, shows a mass loss rather lower at 44.76% than the theoretical mass loss, calculated to be 53.89%, which might be due to SnO_2_ impurities in the sample, although no crystalline SnO_2_ can be seen by XRD. The infrared spectrum of Sn(H‐1,2,4‐BTC) shows the presence of broad O─H stretching bands in the region 2500–3300 cm^−1^, assigned as due to the carboxylic group (Figure S5, Supporting Information). In the carboxylate region the asymmetric and symmetric vibrations of coordinated carboxylates are observed at ≈1620 and ≈1410 cm^−1^, respectively, and also a distinct C═O stretch due to the carboxylic group at ≈1680 cm^−1^ (Figure S6, Supporting Information).

### 2.4 Sn_2_(DOBDC)

**Figure 10:**
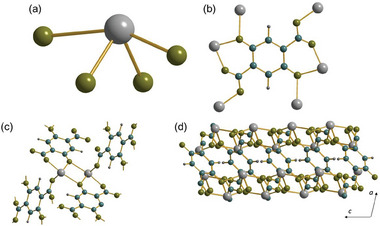
Views of the of structure of Sn_2_(DOBDC) a) the local coordination environment of Sn, b) the binding mode of the DOBDC ligand, c) the dimers of linked Sn centers, and d) the pseudo layered structure. Sn is pale gray, carbon blue, hydrogen dark gray, and oxygen olive.

The ligand DOBDC has been reported by Liu et al. to form a material of composition Sn_2_(DOBDC), prepared in aqueous NaOH at 80 °C.^[^ ^12^ ^]^ We have found that using aqueous KOH as reaction medium at a higher reaction temperature of 130 °C yields a second polymorph of the same composition. The new form of Sn_2_(DOBDC) crystallizes in the monoclinic *P*21/*c* space group and contains one unique Sn(II) cation and a fully deprotonated DOBDC ligand, i.e., all carboxylic and phenolic groups are deprotonated. The Sn(II) has a lone pair, is bonded to four different oxygen atoms, and has again a distorted disphenoidal coordination configuration, **Figure** **10**a. The Sn─O distances range from 2.140(17) Å to 2.20(3) Å with a more distant interaction at 2.60(2) Å, which is as expected for these materials. The smallest bond angle (O2─Sn─O3A) is 83.5(8) ° and the largest bond angle (O2─Sn─O4) is 154.9(4) °. The ligand's two carboxylate groups both bond to the Sn(II) ions in the same way, with a crystallographic center of inversion in the phenyl ring: each of the four carboxylate oxygens on the ligand are bonded to different Sn centers in a *Z*,*E*‐µ_2_‐η^1^:η^1^ binding mode, and each oxido group bridges a pair of tin centers (Figure 10b). While there are direct Sn─O─Sn connections in Sn_2_(DOBDC) these do not connect infinitely, rather are seen in dimeric units, Figure 10c. Although the structure is overall three‐dimensionally connected, it presents a pseudo‐layered structure in the *bc* plane, Figure 10d, where sheets of the connected dimers are found, connected in the *a* direction by organic linkages.

**Figure 11:**
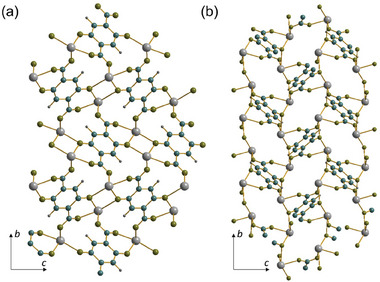
Comparison the of structures of two polymorphs of Sn_2_(DOBDC): a) the material prepared in the current work, with a view perpendicular to the pseudo layered structure showing the connection of the dimer units, and b) the material reported by Liu et al.^[^ ^12^ ^]^ illustrating the infinite inorganic chains. Sn is pale gray, carbon blue, hydrogen dark gray, and oxygen olive.

**Figure** **11** shows a further view of Sn_2_(DOBDC) and compares the structure with the previously reported polymorph of the same composition.^[^ ^12^ ^]^ While the DOBDC ligand is bound in the same way in the two structures, linked to six different tin centers, the bond angles are different such that this new structure contains only Sn─O─Sn bonding dimers, whereas the previously reported polymorph contains infinite [─Sn─O─Sn─] chains.

**Figure 12:**
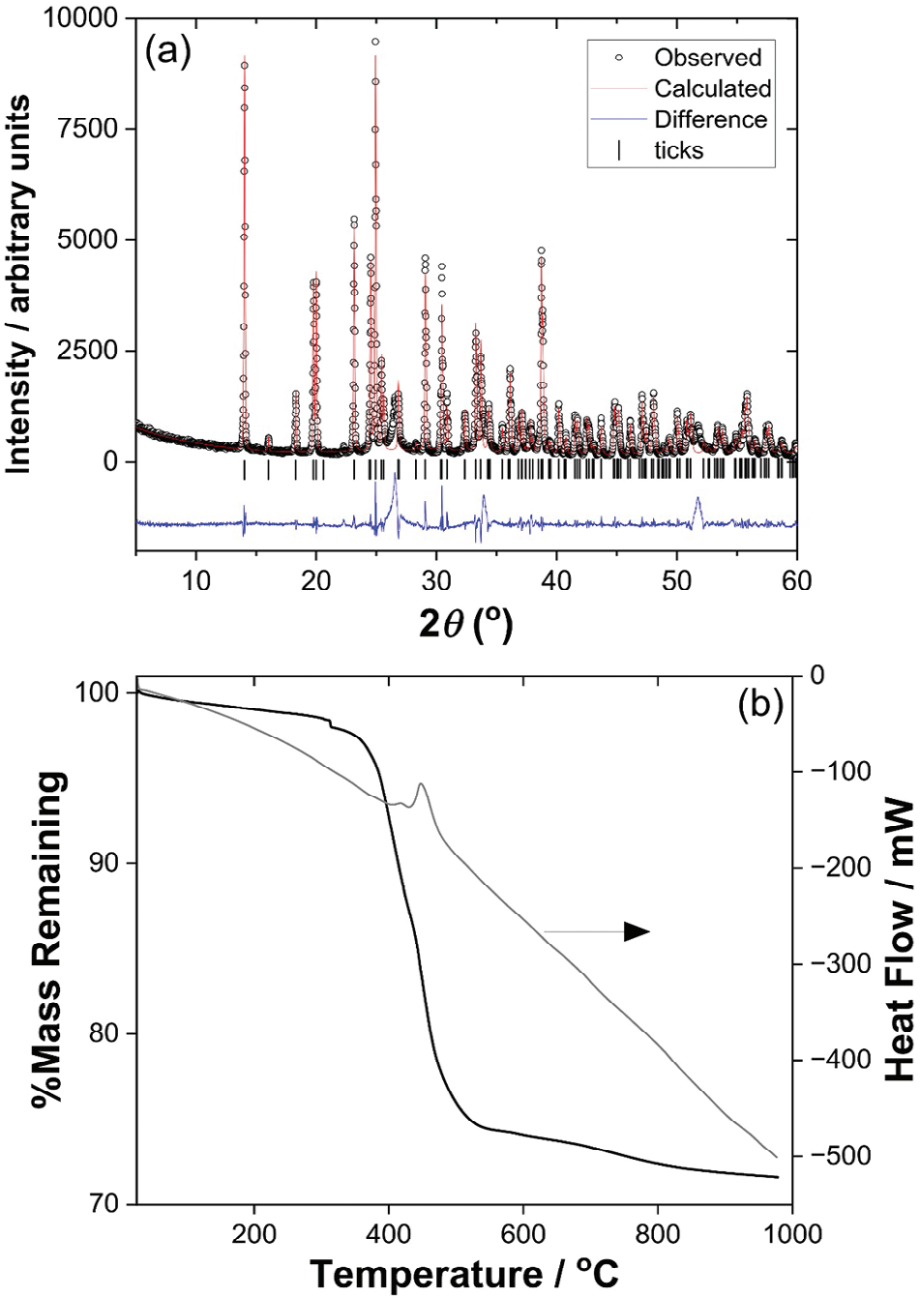
Characterization of the bulk sample of Sn_2_(DOBDC). a) Pawley fitted powder XRD (*λ* = 1.5418 Å, see Supporting Information for fitted lattice parameters) and b) TGA.

**Figure** **12**a shows powder XRD of Sn_2_(DOBDC), which shows good agreement with the simulated pattern from the crystal structure, with evidence for only a trace of SnO_2_ impurity. The TGA, Figure 12b, shows only a slightly smaller mass loss (28.42%) compared to the expected value (30.15%) consistent with the small amount of SnO_2_ present in the sample. The infrared spectrum of Sn_2_(DOBDC) shows no features due to O─H stretches, unlike the other materials discussed so far, which is entirely consistent with the proposed formulation (Figure S7, Supporting Information,) In the carboxylate region the asymmetric and symmetric vibrations of coordinated carboxylates are observed at ≈1530 and ≈1390 cm^−1^, respectively (Figure S8, Supporting Information).

## 3 Conclusions

The structure solution of four coordination polymers of Sn(II) has been possible from microcrystals using synchrotron and electron diffraction techniques, for which conventional single crystal analysis would not be possible in the laboratory. This has allowed a full structural description of new materials that have structures and properties that might be exploited in fields ranging from lithium‐ion storage to pollutant adsorption, especially bearing in mind their stability in water. The materials’ moderate thermal stabilities, to around at least 300 °C, is also of note. The new metal‐organic framework Sn_2_(1,3,5‐BTC)(OH) would be of particular interest for future study given its three‐dimensionally extended structure. Our work illustrates the complexity in the design of new coordination polymers, with polymorphism, inclusion of framework hydroxide ions and partial ligand deprotonation possible depending on only small variations in preparative conditions. The polymorphism we observe for the Sn(II) materials is in part due to the asymmetric coordination of the metal cation with its 5s^2^ electron pair: this means that the local coordination varies between the structures observed. This is different to other cases of polymorphism in other coordination polymers, such as in the zeolite imizadolate family, where the same local metal coordination is found, but the connectivity between the building units defines the long‐range structure. We note that formation of the new polymorphs we have reported here arises from varying solvent or spectator ion in solution, but it would be interesting in future work to consider if interconversion of the polymorphs is possible. The development of crystallographic methods for rapid solution of microcrystals is crucial to understand the huge structural variety in coordination polymers.

## 4 Experimental Section

### Materials Synthesis

Sn_2_(1,3,5‐BTC)OH was prepared by adding SnSO_4_ (0.10 g, 0.47 mmol), LiOH^.^H_2_O, (0.03 g, 0.70 mmol), H_3_‐1,3,5‐BTC (0.19 g, 0.92 mmol) to water (2 mL) and methanol (4 mL) in a 20 mL Teflon autoclave liner. The mixture was stirred for 45 min and placed in an autoclave for 72 h at 170 °C. The reaction was cooled at 1 °C per minute, then the solid product recovered by filtration and washed using water, DMF and acetone before drying in air. The recovered yield of the solid was 46.5% based on SnSO_4_ as the limiting reagent.

Sn(H‐1,3,5‐BTC) was prepared by adding SnSO_4_ (0.30 g, 1.40 mmol), KOH, (0.12 g, 2.14 mmol), H_3_‐1,3,5‐BTC (0.58 g, 2.75 mmol) to water (6 mL) in a 20 mL Teflon autoclave liner. The mixture was stirred for 45 min and placed in an autoclave for 72 h at 170 °C. The reaction was cooled at 10 °C per hour, then the solid product recovered by filtration and washed using water, DMF and acetone before drying in air. The recovered yield of the solid was 29.1% based on SnSO_4_ as the limiting reagent.

Sn(H‐1,2,4‐BTC) was prepared by adding SnSO_4_ (0.30 g, 1.40 mmol), KOH, (0.12 g, 2.14 mmol), H_3_‐1,2,4‐BTC (0.58 g, 2.75 mmol) to water (6 mL) in a 20 mL Teflon autoclave liner. The mixture was stirred for 45 min and placed in an autoclave for 72 h at 180 °C. The reaction was cooled at 10 °C per hour, then the solid product recovered by filtration and washed using water, DMF and acetone before drying in air. The recovered yield of the solid was 42.7% based on SnSO_4_ as the limiting reagent.

Sn_2_(DOBDC) was made by adding SnSO_4_ (0.10 g, 0.47 mmol), KOH, (0.03 g, 0.70 mmol), H_4_DOBDC (0.06 g, 0.30 mmol) to water (6 ml) in a 20 mL Teflon autoclave liner. The mixture was stirred for 60 min and placed in an autoclave for 60 h at 130 °C. The reaction was cooled at 10 °C per hour, then the solid product recovered by filtration and washed using water, DMF and acetone before drying in air. The recovered yield of the solid was 72.7% based on SnSO_4_ as the limiting reagent.

### Structure Determination

Single‐crystal X‐ray diffraction data sets were collected using synchrotron radiation *λ* = 0.6889 Å at beamline I19, Diamond Light Source, UK.^[^ ^24^ ^]^ The beamline was equipped with a Pilatus 2 M detector, and the data were collected at a temperature of 100 K. The data were indexed and integrated using CrysAlisPRO (version 1.171.42.84a). The structures were solved using SHELXT^[^ ^25^ ^]^ and refined with SHELXL,^[^ ^26^ ^]^ implemented using Olex2.^[^ ^23^ ^]^ For both crystals studied, full anisotropic refinement of non‐hydrogen atoms proceeded without need for restraints. Hydrogen atoms were placed at geometrically constrained positions with riding isotropic displacement parameters, with the exception for the hydroxyl proton in Sn(1,3,5‐BTCOH) which was found in the difference map and refined in the presence of a distance restraint [O─H = 0.84(2) Å]. Complete experimental and refinement information are contained in the deposited CIFs along with structure factors and embedded. RES files.

For the 3DED experiment, samples were ground lightly between two glass slides and mounted onto copper‐supported graphene oxide grids to load via a Gatan Elsa cryo holder into a Rigaku XtaLAB Synergy‐ED electron diffractometer, operated at 200 kV and equipped with a Rigaku HyPix‐ED hybrid pixel array area detector. For each sample, data for multiple crystallites (see Table 1) of the order of 1 µm size were collected using selected area continuous rotation electron diffraction at 150(2) K over tilt ranges of ≈100 °. These datasets were individually indexed and integrated to a resolution of 0.8 Å before being scaled and merged into single datasets per sample using CrysAlisPRO (version 1.171.43.95a (Rigaku Oxford Diffraction, 2023)). The structures were solved using ShelXT^[^ ^25^ ^]^ and refined in the kinematic approximation using Olex2.refine as implemented in Olex2,^[^ ^23^ ^]^ version 1.5‐ac6‐012 (compiled 2023.08.24 svn.re1ec1418 for Rigaku Oxford Diffraction, GUI svn.r6817) using published scattering factors.^[^ ^27^ ^]^ In both cases, an extinction correction (as implemented in the Olex2 EXTI command) was applied to decrease the impact of dynamical effects. Further omission of particularly outlying reflections took place with the presumption that they be particularly affected by multiple scattering events. Hydrogens were placed at geometrically constrained positions at neutron distances with riding isotropic displacement parameters, except for the proton engaged in ligand‐ligand hydrogen bonding of Sn(1,2,4‐BTC) which was located in the difference map and refined in the presence of the distance restraint [O─H = 0.90(2) Å]. Complete experimental and refinement information contained in the deposited CIFs along with structure factors and embedded. RES files.

### Bulk Sample Characterization

Powder X‐ray diffraction data were collected using a 3^rd^ generation Malvern Panalytical Empyrean equipped with a multicore (iCore/dCore) optics and a Pixcel3D detector operating in 1D receiving slit mode. A Cu tube was used giving Cu Kα_1/2_ radiation (1.5418 Å). Data were collected between 2ϴ, 3° −60°, using a step size of 0.0131°, and counting time of 2.5 s per step. The diffraction profile was analyzed using the Pawley method with the TOPAS software (academic version 6).^[^ ^28^ ^]^

TGA was performed using a Mettler Toledo TGA/DSC 1 instrument. TGA was used to evaluate the mass loss of samples once heated from room temperature to 1000 °C. 5 mg of sample was weighed into a 40 mL alumina crucible and heated from 25 to1000 °C at a rate of 10 °C per minute. Samples were heated under air flow with a flow rate of 50 mL per minute. Infrared spectra were recorded using a Bruker Alpha II FT‐IR spectrometer with Platinum ATR module.

## Conflict of Interest

The authors declare no conflict of interest.

## Supporting information

Supporting Information

## Data Availability

For the purpose of open access, the author has applied a Creative Commons Attribution (CC‐BY) licence to any Author Accepted Manuscript version arising from this submission. CCDC 2309733‐2309736 contain the supplementary crystallographic data for this paper, available at https://www.ccdc.cam.ac.uk/. Other experimental data are available from https://wrap.warwick.ac.uk/81432.

## References

[1] H. Furukawa , K. E. Cordova , M. O'Keeffe , O. M. Yaghi , Science 2013, 341, 1230444. 23990564 10.1126/science.1230444

[2] R. Freund , O. Zaremba , G. Arnauts , R. Ameloot , G. Skorupskii , M. Dincă , A. Bavykina , J. Gascon , A. Ejsmont , J. Goscianska , M. Kalmutzki , U. Lächelt , E. Ploetz , C. S. Diercks , S. Wuttke , Angew. Chem., Int. Ed. 2021, 60, 23975. 10.1002/anie.202106259 33989445

[3] V. F. Yusuf , N. I. Malek , S. K. Kailasa , ACS Omega 2022, 7, 44507. 36530292 10.1021/acsomega.2c05310 PMC9753116

[4] Tin Chemistry: Fundamentals, Frontiers, and Applications (Eds: M. Gielen , A. G. Davies , K. Pannell , E. Tiekink ), John Wiley & Sons, Inc., Hoboken, New Jersey 2008.

[5] P. Y. Dapsens , C. Mondelli , J. Pérez‐Ramírez , Chem. Soc. Rev. 2015, 44, 7025. 25917850 10.1039/c5cs00028a

[6] C. G. dos Santos , G. M. de Lima , Coord. Chem. Rev. 2020, 410, 213236.

[7] G. M. de Lima , R. I. Walton , G. J. Clarkson , R. S. Bitzer , J. D. Ardisson , Dalton Trans. 2018, 47, 8013. 29869660 10.1039/c8dt01370e

[8] S. Yuan , L. Feng , K. Wang , J. Pang , M. Bosch , C. Lollar , Y. Sun , J. Qin , X. Yang , P. Zhang , Q. Wang , L. Zou , Y. Zhang , L. Zhang , Y. Fang , J. Li , H.‐C. Zhou , Adv. Mater. 2018, 30, 1704303. 10.1002/adma.201704303 29430732

[9] M. Ding , X. Cai , H.‐L. Jiang , Chem. Sci. 2019, 10, 10209. 32206247 10.1039/c9sc03916c PMC7069376

[10] Z. Chen , K. O. Kirlikovali , L. Shi , O. K. Farha , Mater. Horiz. 2023, 10, 3257. 37285170 10.1039/d3mh00541k

[11] A. Ghosh , S. Gumma , G. Das , J. Photochem. Photobiol. A. 2020, 403, 112863.

[12] J. Liu , D. Xie , X. Xu , L. Jiang , R. Si , W. Shi , P. Cheng , Nat. Commun. 2021, 12, 3131. 34035247 10.1038/s41467-021-23335-1 PMC8149848

[13] S.‐B. Xia , L.‐F. Yao , H. Guo , X. Shen , J.‐M. Liu , F.‐X. Cheng , J.‐J. Liu , J. Power Sources 2019, 440, 227162.

[14] J. Jiang , R. Zhang , T. Sun , J. Guo , J. Liu , P. Cheng , W. Shi , Inorg. Chem. 2023, 62, 16609. 37767995 10.1021/acs.inorgchem.3c02699

[15] J. Liu , J. Jiang , Q. Zhou , Z. Chen , R. Zhang , X. Xu , X. Han , S. Yang , Z. Zhou , P. Cheng , W. Shi , eScience 2023, 3, 100094.

[16] L. Ding , X. Jiang , K. Li , J. Wen , M. Zeng , Electrochim. Acta 2023, 464, 142901.

[17] H.‐X. Qi , H. Jo , X. Chen , J. Hong , K. M. Ok , Inorg. Chem. 2020, 59, 11554. 32568526 10.1021/acs.inorgchem.0c01358

[18] N. E. Brese , M. O'Keeffe , Acta Crystallogr., Sect. B: Struct. Sci., Cryst. Eng. Mater. 1991, 47, 192.

[19] A. K. Cheetham , C. N. R. Rao , R. K. Feller , Chem. Commun. 2006, 4780. 10.1039/b610264f 17345731

[20] C. F. Macrae , I. Sovago , S. J. Cottrell , P. T. A. Galek , P. McCabe , E. Pidcock , M. Platings , G. P. Shields , J. S. Stevens , M. Towler , P. A. Wood , J. Appl. Cryst. 2020, 53, 226. 32047413 10.1107/S1600576719014092 PMC6998782

[21] P. R. Spackman , M. J. Turner , J. J. McKinnon , S. K. Wolff , D. J. Grimwood , D. Jayatilaka , M. A. Spackman , J. Appl. Cryst. 2021, 54, 1006. 34188619 10.1107/S1600576721002910 PMC8202033

[22] W. H. Baur , A. A. Khan , Acta Crystallogr., Sect. B: Struct. Sci., Cryst. Eng. Mater. 1971, 27, 2133.

[23] O. V. Dolomanov , L. J. Bourhis , R. J. Gildea , J. A. K. Howard , H. Puschmann , J. Appl. Cryst. 2009, 42, 339. 10.1107/S0021889811041161 PMC3236671 22199401

[24] H. Nowell , S. A. Barnett , K. E. Christensen , S. J. Teat , D. R. Allan , J. Synchrotron Radiat. 2012, 19, 435. 22514182 10.1107/S0909049512008801

[25] G. M. Sheldrick , Acta Crystallogr., Sect. A: Found. Adv. 2015, 71, 3. 25537383 10.1107/S2053273314026370 PMC4283466

[26] G. Sheldrick , Acta Crystallogr., Sect. A: Found. Adv. 2008, 64, 112. 10.1107/S0108767307043930 18156677

[27] A. Saha , S. S. Nia , J. A. Rodríguez , Chem. Rev. 2022, 122, 13883. 35970513 10.1021/acs.chemrev.1c00879 PMC9479085

[28] A. A. Coelho , J. Appl. Cryst. 2018, 51, 210.

