# Supplementary material for: Polymorphism and Structural Variety in Sn(II) Carboxylate Coordination Polymers Revealed from Structure Solution of Microcrystals
Source: Small Methods. 2024 Mar 10;9(2):2301703. doi: 10.1002/smtd.202301703 (PMC11843403; doi:10.1002/smtd.202301703)
Supplement: Supplementary file 1 — Supporting Information [file SMTD-9-2301703-s001.pdf]

small methods

Supporting Information

for *Small Methods*, DOI 10.1002/smtd.202301703

Polymorphism and Structural Variety in Sn(II) Carboxylate Coordination Polymers Revealed from Structure Solution of Microcrystals

*Avneet K. Ramana, Jeremiah P. Tidey, Geraldo M. de Lima and Richard I. Walton**

Supporting Information

Polymorphism and Structural Variety in Sn(II) Carboxylate Coordination Polymers Revealed from Structure Solution of Microcrystals

Avneet K. Ramana, Jeremiah P. Tidey, Geraldo M. de Lima, Richard I. Walton*

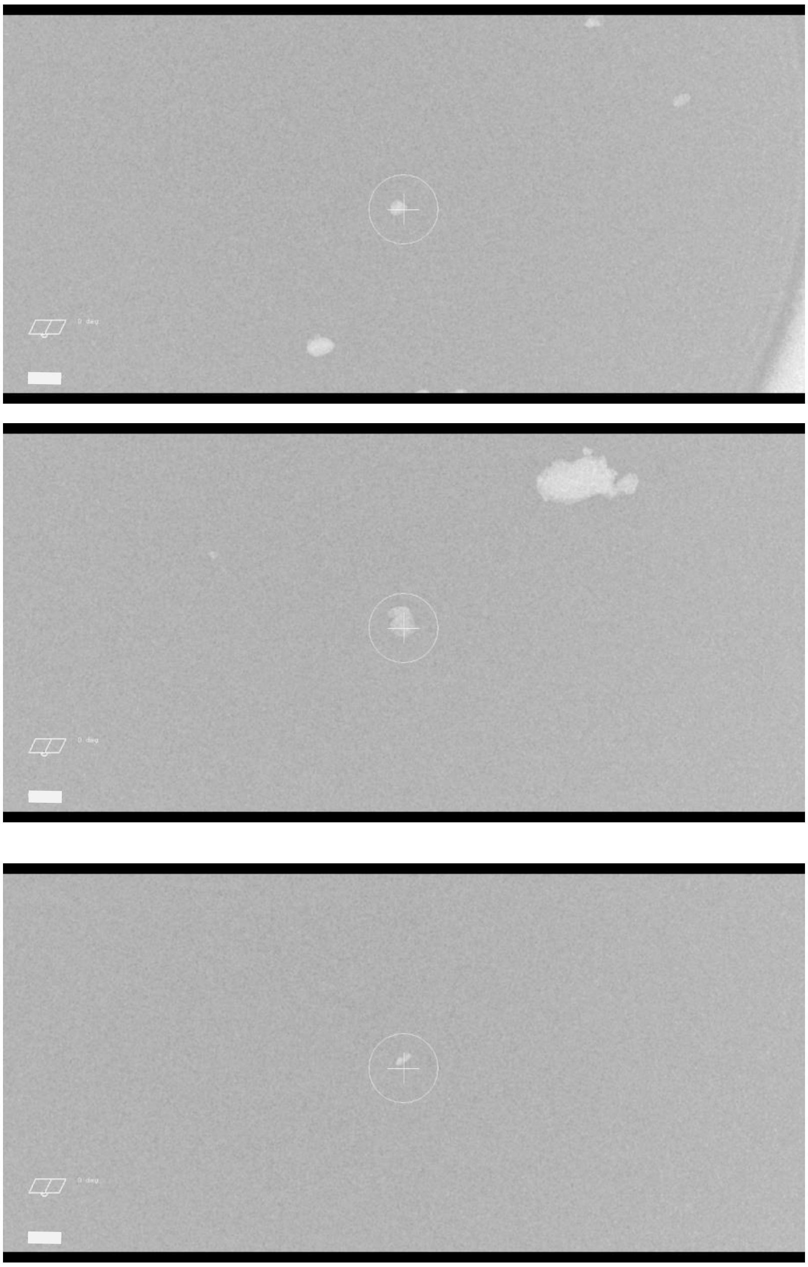

Figure S1: Images of 3 crystals of Sn(H-1,2,4-BTC) studied by 3DED, as indicated by the circled region. The scale bar for each is 1 μm.

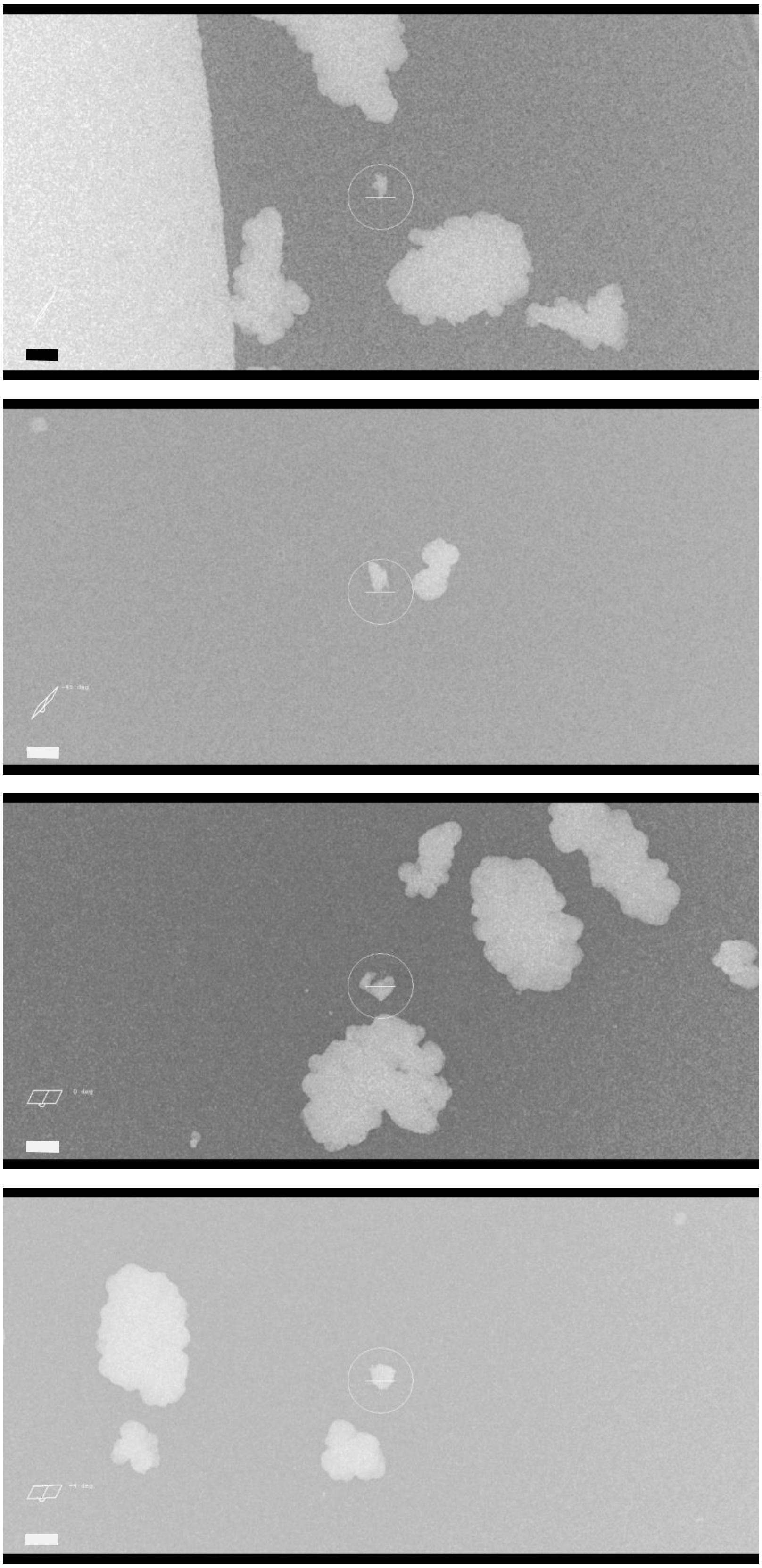

Figure S2: Images of 4 crystals of Sn_2(DOBDC) studied by 3DED, as indicated by the circled region. The scale bar for each is 1 μm.

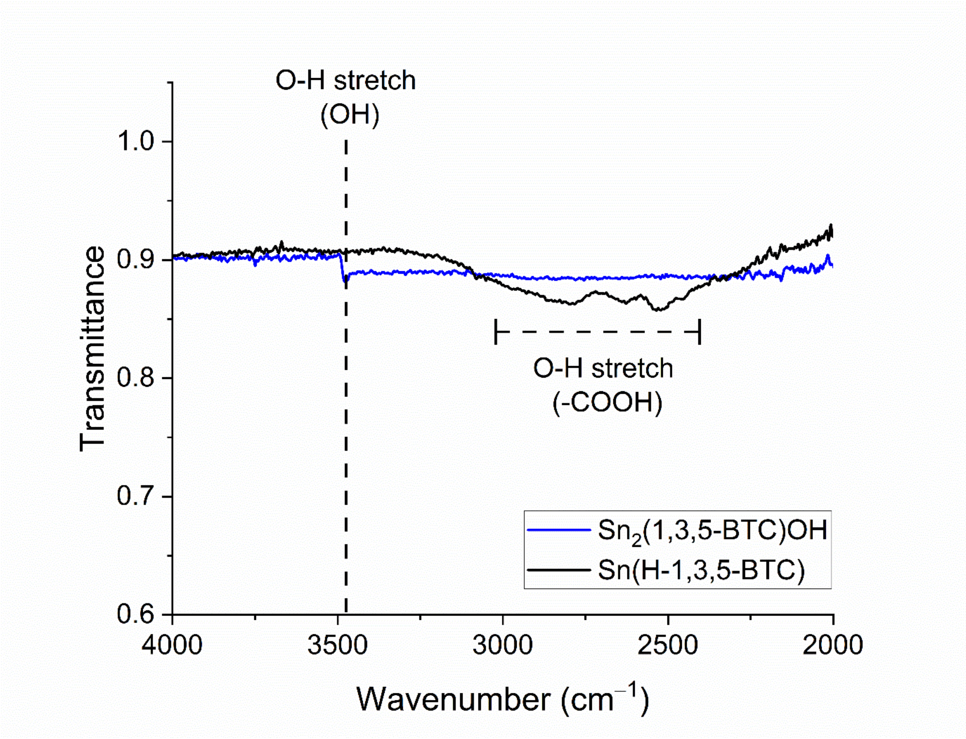

Figure S3: IR spectra in the O-H stretch region of $Sn_2(1,3,5\text{-}BTC)(OH)$ and Sn(H-1,3,5-BTC).

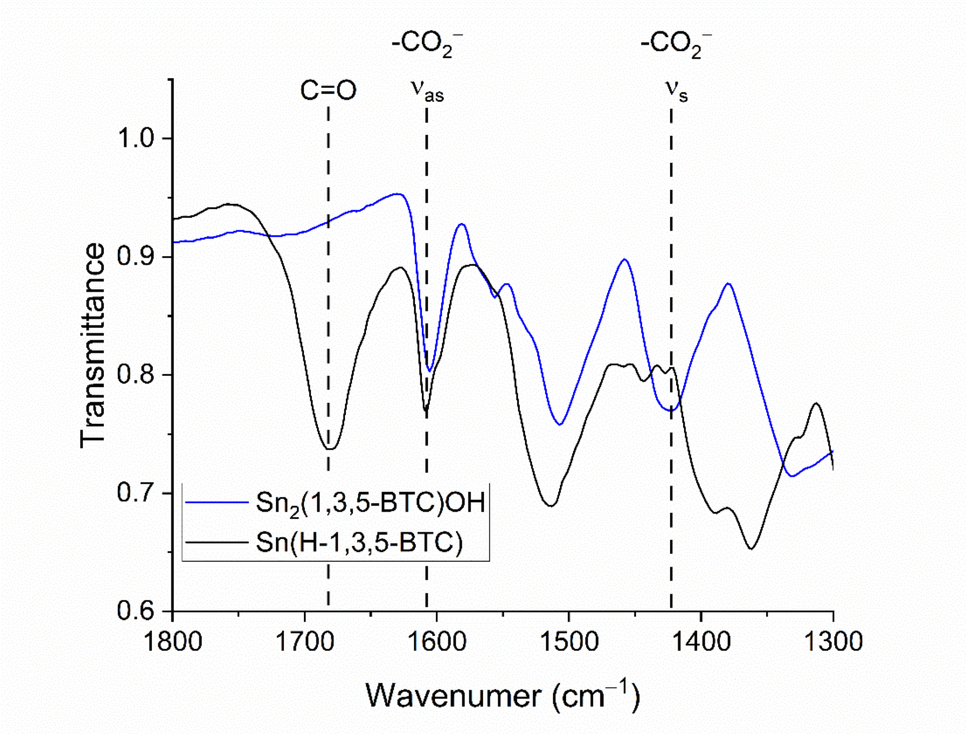

Figure S4: IR spectra in the carboxylate region of $Sn_2(1,3,5\text{-}BTC)(OH)$ and Sn(H-1,3,5-BTC).

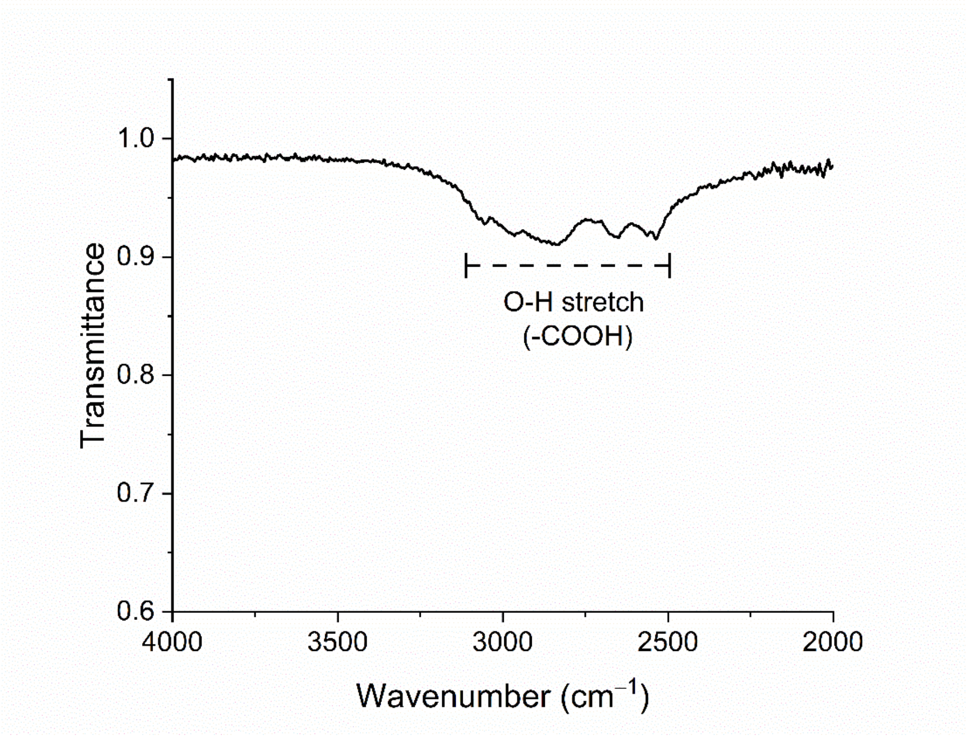

Figure S5: IR spectrum in the O-H stretch region of Sn(H-1,2,4-BTC)

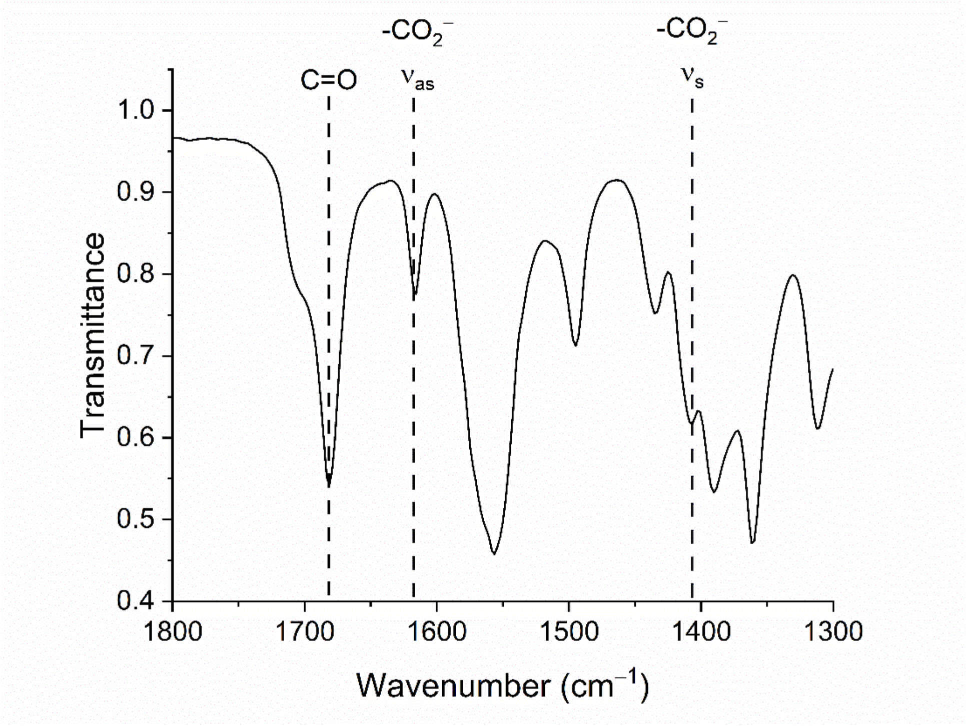

Figure S6: IR spectrum in the carboxylate region of Sn(H-1,2,4-BTC)

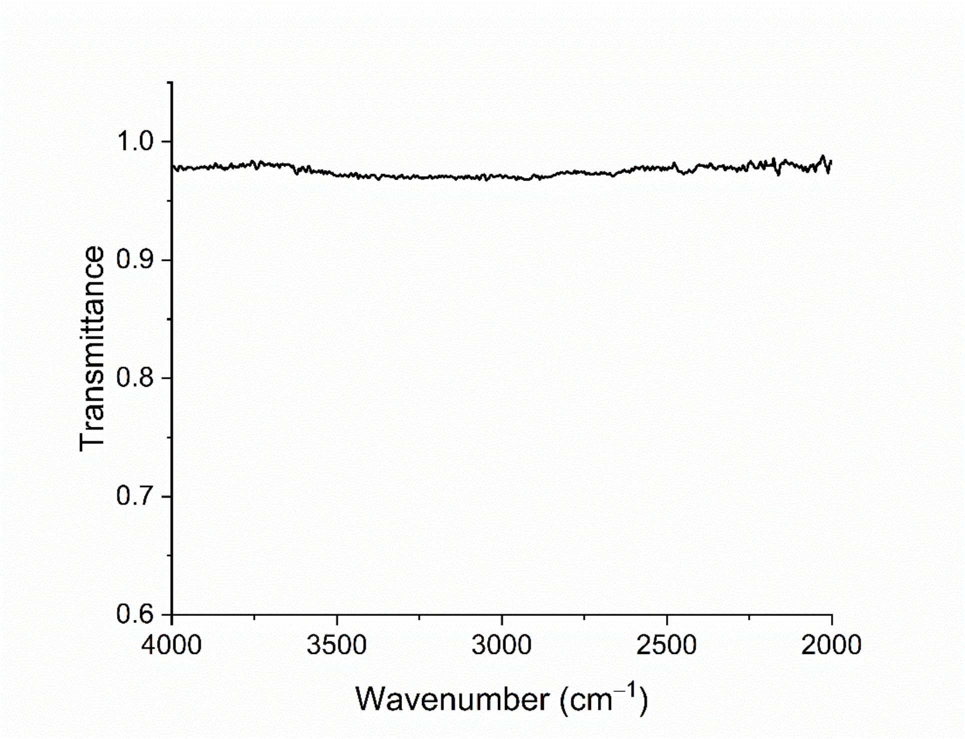

Figure S7: IR spectrum in the O-H stretch region of Sn_2(DOBDC).

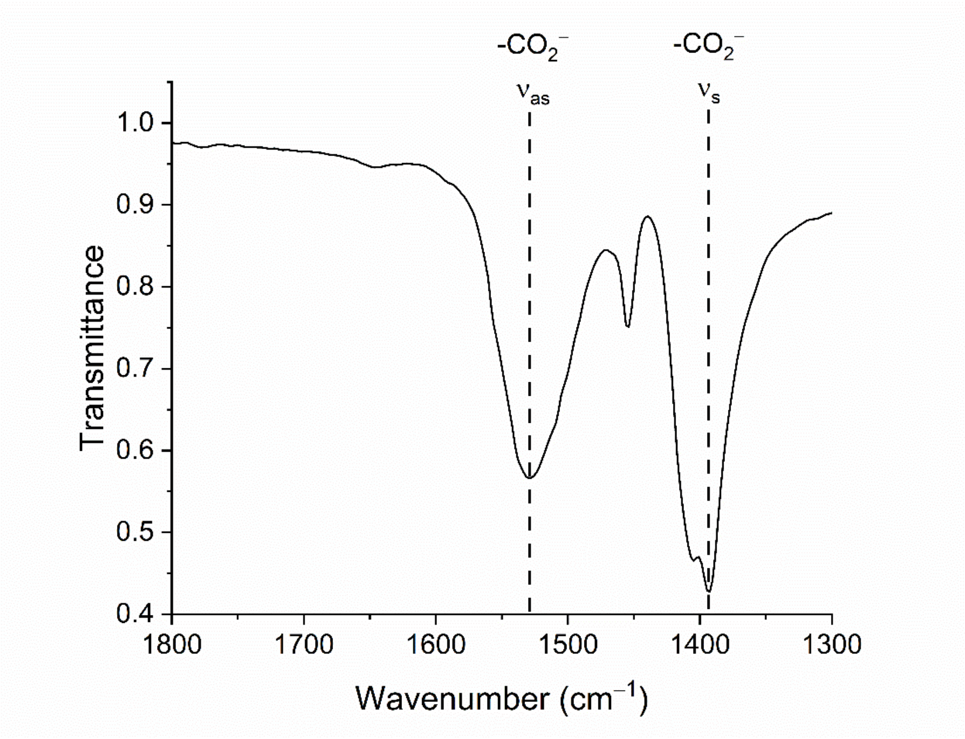

Figure S8: IR spectrum in the carboxylate region of Sn_2(DOBDC).

Table S1: Comparison of lattice parameters from single crystal structure determination and from Pawley refined against powder XRD patterns for Sn_2(1,3,5-BTC)(OH). Space group: $Pna2_1$.

	**Single Crystal (100 K)**	**Pawley refinement (293 K)**
a/Å	10.7763(5)	10.8260(3)
b/Å	12.4014(6)	12.4529(5)
c/Å	7.9574(3)	8.0519(2)
V/Å^3	1063.44(8)	1085.53(7)
R_{wp} / %	-	20.1

Table S2: Comparison of lattice parameters from single crystal structure determination and from Pawley refined against powder XRD patterns for Sn(H-1,3,5-BTC). Space group: $P\overline{1}$.

	**Single Crystal (100 K)**	**Pawley refinement (293 K)**
a/Å	4.5534(3)	4.5985(7)
b/Å	9.8583(7)	9.8952(8)
c/Å	10.3440(8)	10.4302(8)
α/°	72.621(7)	73.361(5)
β/°	84.440(6)	85.566(10)
γ/°	79.459(6)	79.58(1)
V/Å^3	435.21(6)	447.07(9)
R_{wp} / %	-	13.4

Table S3: Comparison of lattice parameters from single crystal structure determination and from Pawley refined against powder XRD patterns for Sn(H-1,2,4-BTC). Space group: *C*2/*c*.

	**Single Crystal (150 K)**	**Pawley refinement (293 K)**
a/Å	13.90(9)	14.0099(7)
b/Å	4.87(10)	4.8884(2)
c/Å	25.65(19)	25.915(1)
β/°	97.33(16)	97.613(3)
V/Å^3	1722(39)	1759.2(1)
R_{wp} / %	-	9.0

Table S4: Comparison of lattice parameters from single crystal structure determination and from Pawley refined against powder XRD patterns for Sn_2(DOBDC). Space group: $P2_1/c$.

	**Single Crystal (150 K)**	**Pawley refinement (293 K)**
a/Å	4.98(7)	5.0128(2)
b/Å	11.05(5)	11.0573(4)
c/Å	7.92(10)	7.9488(3)
β/°	104.6(6)	104.905(2)
V/Å^3	422(8)	425.77(3)
R_{wp} / %	-	16.02
